# Transition to Preschool: Paving the Way for Preschool Teacher and Family Relationship-Building

**DOI:** 10.1007/s10566-023-09735-y

**Published:** 2023-01-31

**Authors:** Martina Andersson Søe, Elinor Schad, Elia Psouni

**Affiliations:** Department of Psychology, Lund University, Box 213, S-221-00 Lund, Sweden

**Keywords:** Preschool transition, Preschool, Childcare, Child–teacher interaction, Parent–teacher interaction, Child attachment development, Sweden

## Abstract

**Background:**

Previous research suggests that interactions between preschool teachers and children in early care and educational contexts can contribute to the child’s positive attachment development and socioemotional adjustment.

**Objective:**

Investigate how the transition process to preschool is organized and whether various ways of organizing it may differently influence family–teacher relationship-building and child adjustment.

**Methods:**

Conducted a mixed methods study of quantitative and qualitative survey data from Swedish preschool professionals (*N* = 535).

**Results:**

Preschool introduction varied across preschools in several structural aspects such as introduction length and intensity, timing for first child–parent separation, and number of children and teachers involved in the introduction process. Results moreover suggested that different introduction models were associated with different ways of engaging the parent, where the “parent-active” model was characterized by a high level of parental participation during the introductory activities. This was perceived by preschool professionals as positively influencing the family–teacher relational formation.

**Conclusion:**

Findings suggest that inviting parents to participate actively in preschool transition may help better engage them in the introduction process, which in turn may positively influence family–teacher relationship-building. Future research should focus in more detail on how child–teacher and parent–teacher interactions, respectively, influence family–teacher relationship-building and child adjustment during, and after, the introduction period.

## Introduction

Since the emergence of attachment theory (Bowlby, 1969/1982, 1973), developmental and educational psychologists have regarded the quality of caregiver–child interactions in the early care environment as crucial for child development. How parents respond to and regulate a child’s need for emotional security influences the child’s development of the ability to cope with emotional stress as well as what they expect of others in future social interactions. Indeed, the quality of child–parent early attachment interactions is predictive of children’s later socioemotional development, including peer relationships, social competence, and behavioral problems (Groh et al., 2017; Psouni et al., 2015). Besides parents, children also form relationships with other regularly available caregivers, such as preschool teachers. Since preschool is a central component in many children’s everyday life, several studies have investigated the interaction quality between children and teachers at preschool and found that these relationships may also influence children’s attachment development (e.g., Ahnert et al., 2006; Howes & Spieker, 2016) and later academic outcomes (Ulferts et al., 2019). Thus, enabling a good foundation for the development of child–teacher relationships in preschool is important.

Preschool teachers regard relational engagement with children as central to their work, both in Sweden (Broman & Persson, 2018) and internationally (McNally & Slutsky, 2018). Not much is empirically known, however, about how to organize preschool everyday life to support the emergence of strong child–teacher relationships, and one Swedish study showed that teachers in general have little consensus on the conceptualization and implementation of relational engagement (Broman & Persson, 2018). This lack of consensus is associated with lower preschool care quality (The Swedish Schools Inspectorate, 2018), perhaps because teachers’ individual views of child behavior (Degotardi, 2010) and their profession (Thomason & La Paro, 2013) influence how they interact with children during daily preschool activities. An unclear understanding of relational engagement might be especially problematic during the child’s transition to preschool, as building a foundation for the relationships between teachers and children is essential during this period (e.g., Brooker 2008; Ebbeck & Yim, 2009). However, there is little empirical basis on how best to organize this introduction phase. Thus, from the perspective of preschool teachers, the aim of this study was to identify potentially important organizational features of the preschool introduction process that may foster relationship-building between teachers and children.

### The Importance of Interactions at Preschool

Parent–child attachment bonds are generally stronger than the bonds children form with preschool teachers (Verschueren & Koomen, 2012). Still, interactions with preschool teachers can contribute to children’s socioemotional (e.g., Beckh & Becker-Stoll 2016; Grossmann et al., 2005; Sagi-Schwartz & Aviezer, 2005) and academic (Ulferts et al., 2019) development. Furthermore, child attachment development goes beyond the dyadic interactions with parental attachment figures. Introducing ecological (Bronfenbrenner, 1979) and (family) systemic (e.g., Minuchin 1985) perspectives into the attachment theory framework suggests that the relational dynamics of different significant caregivers (Rothbaum et al., 2002) in different settings (Sabol & Pianta, 2012) are uniquely important for children’s adjustment and further development. Indeed, a recent study (Lang et al., 2020) revealed that the interactions between the child’s parent(s) and preschool teachers are comparable to “coparenting” interactions (Feinberg, 2003), which are known to impact different aspects of children’s socioemotional development (Psouni, 2019; Teubert & Pinquart, 2010). Accordingly, a child’s adjustment to preschool may depend not only on interactions between preschool teachers and the child but also on those between teachers and parents.

### Transition to Preschool: Establishing New Interactions

Given that the quality of relations between children, parents, and preschool teachers contributes to a child’s preschool adjustment and further development, it is important to ensure that organization of preschool activities supports good quality interactions. The preschool introduction process has particular significance here. First, this transition bridges the developmental contexts of family everyday life and preschool (e.g., Brooker 2008). Second, the process is emotionally stressful for children, as indicated by elevated cortisol levels in children during preschool enrollment (Ahnert et al., 2004; Bernard et al., 2015; Nystad et al., 2021). This makes sense, according to attachment theory, as entering preschool not only separates children from their parent(s)—one of the most stressful experiences for small children—but also presents them with an unfamiliar situation.

When in unfamiliar surroundings, children instinctually seek proximity to, and comfort from, a close caregiver to mitigate the resultant emotional distress (Ainsworth et al., 1978; Bowlby, 1969/1982). Indeed, secure child–parent interactions appear to act as buffers against stress in children during the transition process (Ahnert et al., 2004). Regarding child–teacher interactions, one study similarly showed that children who had more reciprocal interactions with preschool teachers during the transition process demonstrated higher levels of exploratory behavior and expressed more positive emotions over time (Datler et al., 2012), suggesting the emergence of a greater emotional security. These effects were independent of age, gender, and temperament (Datler et al., 2012). Thus, stress during preschool transition in young children can be mitigated by the interplay with both parents and teachers.

### Preschool Transition Procedures

According to both systems and attachment theories, the preschool transition process is thus essential for a child’s future adjustment. However, in Nordic countries, there are no guidelines on how to conduct preschool introduction (Daníelsdóttir & Ingudóttir, 2020). The only empirical data on introduction conduct come from a qualitative study of 17 preschool teachers in Sweden (Markström & Simonsson, 2017) and one survey study published locally in Norway (Drugli et al., 2017). Both studies describe preschools as employing a “traditional model” consisting of the child and parent(s) attending preschool only few hours’ per day over about two weeks, or a more recently developed “parent-active model” wherein both child and parent(s) attend the entire educational program (e.g., 09:00–14:00) for between three and five days.

There is nevertheless a lack of large scale, statistical corroboration of different features in ways of introducing children to preschool. This is noteworthy, especially when considering that preschool teachers’ beliefs about their profession, pedagogical structure and discipline, and children’s capacities, impact their interactions with children (Degotardi, 2010; McNally & Slutsky, 2018; Thomason & La Paro, 2013). Thus, different ways of organizing the introduction process may influence the quality of interactions and relationship-formation between preschool teachers, children, and even parents. It is therefore essential to explore how preschools organize different features of the introduction process and how teachers evaluate the effects of the organization method.

### Aims and Study Design

The objective of this study is to provide a comprehensive overview of the features of the preschool introduction process and their potential relevance to family–teacher relationship-building and child adjustment. To this end, we utilized a mixed methods approach combining quantitative assessment of different ways of organizing preschool introduction with qualitative, first-hand accounts from teachers of “what works and why”. This overview aims to offer a necessary step towards an empirically informed foundation for tailoring different features of preschool introduction to benefit children’s and parents’ wellbeing.

This study was conducted in the Swedish preschool system. In Sweden, due to legislation on children’s rights, childcare, and parental leave, most children are introduced to preschool before their second birthday, which is considered a very sensitive period of attachment development (e.g., De Wolff & van Ijzendoorn 1997). Therefore, Sweden offers a unique context for investigating preschool care from the perspective of attachment theory.

Aiming at a large-scale data collection, we collected survey data from preschool teachers at a national level. To secure the desired first-hand preschool teacher perspective, we employed a convergent, mixed methods approach (Creswell & Plano Clark, 2018; Johnson & Turner, 2003) to enable a simultaneous collection of quantitative and qualitative data. The specific aims of the study were as follows:

1. Quantitatively and qualitatively outlining the organization of preschool introduction: how is preschool introduction conducted in Sweden? We expected to be able to identify different procedures and their structural organization.
2. Examine how the different organizational factors during the introduction phase might have implications to family–teacher relational establishment and child adjustment.

Based on the current literature (e.g., Ebbeck & Yim, 2009; Lang et al., 2016; Markström & Simonsson, 2017; McNally & Slutsky, 2018), we expected that different introduction models would imply variations in family–teacher relationship strength and child adjustment.

## Methods and Materials

### Procedure

The study protocol was approved by the Swedish Ethical Review Authority. Pilot data collection and feedback from four preschool teachers of different ages and educational levels suggested high face validity of the survey and guided some minor modifications in item formulations. The finalized survey was administered from January to March 2021 through the online data collection tool RED Cap (Harris et al., 2009). To secure a large sample with good geographical and demographical variance, we used a snowball sampling strategy; we reached out to several online forums for preschool professionals and encouraged them to disseminate the survey in their networks. Data collection was monitored to ensure geographical spread and we directly contacted preschools from underrepresented areas. Participants gave their informed consent electronically. The survey took 10–15 min to complete. To protect participant anonymity, we did not ask for them to name their preschool of employment. Because data collection took place solely online and anonymously, we did not engage with the participants at any point.

### Participants

In total, 710 participants consented to participate. Of those, 103 did not proceed to the survey and an additional 72 dropped out after completing the background information. The sociodemographic characteristics of those who dropped out were no different from the final sample of *N* = 535 preschool teachers.

Participant age ranged from 22 to 71 years (*M* = 45.11 years, *SD* = 10.41), aligning with the age range of the preschool teacher population in Sweden (The Swedish National Agency for Education, 2021). Preschool work experience ranged from 1 to 45 years (*M* = 18.48 years, *SD* = 11.20). Among the participants who reported their gender, 99% were women (corresponding well to the preschool teacher population of 96% women; The Swedish National Agency for Education, 2021). Of those who were employed full-time, 60.83% were licensed preschool teachers, suggesting an overrepresentation compared to the national population (40.40%). About a third of participants worked at preschools in urban areas (38.10%); the rest worked in suburban (32.80%) or rural areas (29.10%). About 83% of participants reported conducting preschool introductions for children as part of their daily work. Those who did not (*n* = 45) were either school leaders (*n* = 21) or special education teachers (*n* = 10).

### Measures

Due to a lack of validated instruments for assessing preschool introduction organization, we developed relevant survey questions for this study. We utilized our expertise in the socioemotional development of young children and our knowledge of attachment theory and systems theory to formulate questions that aligned well with current theory and everyday preschool life. The first author is a female doctoral student in developmental psychology as well as a clinical psychologist with previous placements in preschool and family contexts; the second author is a female senior lecturer in educational psychology with a PhD in work and organizational psychology and experience as a preschool psychologist; the third author is a female professor in developmental psychology and a clinical psychologist with expertise in early childhood and family development. To further ensure contextual sensitivity towards Swedish preschools, we reviewed practitioner literature about preschool introduction (Källhage & Malm, 2020).

#### Structural Organization of the Introduction Phase

To evaluate how the introduction phase was structured, we used 12 questions regarding introduction name, length and intensity of introduction procedure, number of staff involved and children introduced simultaneously, and level of organizational preparation and flexibility. To obtain the teachers’ first-hand accounts, we included the option for open-ended responses when relevant.

The survey began with a dichotomous question “Does your preschool have a specific procedure for how to conduct the introduction of children at your preschool?” (yes/no). This was followed with an open question: “If the introduction phase follows a specific procedure, what do you call this procedure?” Participants were then asked to numerically report the introduction length (days), intensity (hours/day), number of staff responsible for introducing the child, and number of children usually introduced simultaneously. Response alternatives “It varies from time to time” and “Don’t know” were included. Participants were then asked to nominally report whether they first separated the child and parent during the first, second, or third week of the introduction phase, before rating the preparatory work and flexibility of introduction organization (1 = “Not at all”, 5 = “To a high degree”). For instance, they were asked: “To what degree do you adjust the introduction phase according to the needs of the child and the family?” Following survey construction recommendations by Saris and Gallhofer (2007), a neutral middle alternative was included, as well as a sixth alternative of “Don’t know”. They then rated their general satisfaction with the introduction conduct (1 = “Not at all”, 5 = “To a high degree”), which was followed by an open-ended question on sources of satisfaction/dissatisfaction with the specific introduction conduct they used. Finally, they completed four questions on the impact of the COVID-19 pandemic on the introduction conduct, which are discussed elsewhere (Andersson Søe et al., 2022).

### Data Analysis

As per the mixed methods convergent design, we analyzed the quantitative and qualitative data separately before merging the results. The quantitative and qualitative strands were given equal priority (QUAN + QUAL; Plano Clark & Ivankova 2016) because they addressed our research aims in an equally important way. The quantitative analysis was used to identify the structural organization of different preschool introduction models (based on e.g., length, intensity, child-adult ratios, preparation), while the qualitative analysis (e.g., what makes a satisfactory introduction phase) was used to provide first-hand accounts from teachers of “what works and why” during the introduction process. Lastly, we quantified the teachers’ qualitative accounts to merge them into the quantitative overview of what characterizes different preschool introduction models. This mixed methods analysis thus enabled us to integrate an inductive, first-hand teacher-perspective of “what works and why” when comparing the organization of models and, subsequently, when analyzing possible implications of different organizational factors for family–teacher relationship building and child adjustment.

#### Quantitative Analysis

We categorized different introduction procedures named by participants into introduction conduct models (see “Categorization of different introduction models”). Next, we calculated descriptive statistics of the structural characteristics (e.g., introduction length, intensity, child–adult ratios, preparation) of each introduction model. We compared the models using Pearson’s Chi-square tests for nominal data or non-parametric tests (Kruskal-Wallis or Mann-Whitney U) for continuous data because the data were non-normally distributed and the group sizes of introduction models were uneven. To avoid the risk of type I errors in multiple comparative tests, significant values were Bonferroni corrected.

#### Qualitative Analysis

Qualitative data derived from written survey text can be less dense than, for instance, interview data, so we adopted content analysis (Graneheim & Lundman, 2004) to analyze the open-ended responses of what made them satisfied or dissatisfied with the introduction procedure. With content analysis, we could attend to the manifest content of the data (i.e., its descriptive elements) while also analyzing its underlying meaning (i.e., focusing on its latent content; Graneheim & Lundman 2004). This descriptive, yet interpretative approach, thus enabled meaningful quantification of the relative frequency of the themes (Guest et al., 2012). Free responses comprised in total 44 pages of transcript, contributed by 413 participants. The sociodemographic characteristics (see “Participants”) did not differ between participants who provided open-ended data and those who did not (*n* = 123).

Line-by-line coding was employed as the unit of analysis to secure a high level of abstraction (Graneheim & Lundman, 2004). Attachment theory, systems theory, and empirical knowledge about preschool introduction were used to deductively scaffold the analysis; however, we also inductively coded responses that deviated from the theoretical framework—so-called “left-over data” (Graneheim et al., 2017)—to refine the analysis. All data were thus considered in the coding and categorization process. After a thorough first read, recurring words and sentences describing coherent or similar phenomena were noted as initial meaning units. We then analyzed these in their context to abstract their latent meaning and labelled them with codes (see “Descriptive themes of preschool teachers’ qualitative accounts” for code examples) for theme categorization. We extracted six descriptive themes and eight sub-themes along with their frequencies in the text.

To secure the credibility of the qualitative analysis, we followed Graneheim and Lundman’s (2004) approach to dialogue between researchers rather than relying merely on coding consensus. The first author therefore took primary responsibility for the coding process, but all authors were engaged in repeated and extensive discussions on theme development. Disagreements were used to inform and enrich the process. Thus, our results express a dynamically developed agreement between the authors on how to interpret the data. Credibility was further enhanced by generous use of quotes (Graneheim & Lundman, 2004). As our mixed methods analysis required quantification of our qualitative results, we also conducted an interrater reliability check: about 30% of codable data fragments were coded for main theme categorization by the second and third author, blind to the first author’s coding. The interrater reliability was 90% (κ **=** 0.87; *p* < .001).

#### Mixed Methods Analysis

To explore how the qualitative findings related to specific introduction models, we used the mixed methods integration strategy of transforming the frequency data from the content analysis into quantitative variables (Creswell & Plano Clark, 2018). We then ran Pearson’s Chi-square tests to compare the distribution of theme frequency between the introduction models. Only participants who contributed with both quantitative and qualitative data were included in this analysis (*n* = 413). The implications of these integrative results, i.e., meta-inferences (Creswell & Plano Clark, 2018), for family–teacher relationship-building and child adjustment were included in the discussion.

## Results

Among participants, 86% (*n* = 460) reported following a specific model of introduction. Of those, almost all (98%) reported that the model was known to all teachers in the team.

### Categorization of Different Introduction Models

About 80% of responses on introduction procedures included either the term “parent-active” or similar or just the word “introduction”. Based on the categorization by Markström and Simonsson (2017), most responses could thus be classified as either “parent-active model” (PAM) or “traditional model” (TM). Some respondents (*n* = 22) added “10 days”, “15 days”, or “long” to “introduction”; we classified these as TM since Markström and Simonsson (2017) described that model as commonly lasting two weeks or more. Seventy-five participants responded that their preschool had no specific introduction procedure, which we termed “undefined model” (UM).

Of the remaining 20% of responses, 24 included expressions such as “attachment”, “best for child”, “child in the center”, “relationally based”, “individual process”, or “family–preschool interplay”, which could not be classified by their names. Eighty-five responses did not name the introduction procedure. Preliminary group comparison of the structural features of the introduction process revealed that these 109 responses differed from the PAM in several respects (e.g., length, intensity, and number of teachers and children involved in the introduction process) but not from the TM. These responses were therefore classified as the TM, resulting in a final categorization of three introduction models (see Table 1 for descriptive overview).

**Table 1:** N, Means, Standard Deviations, Minimum and Maximum Values of Organizational Aspects of Introduction Conduct

Structural Aspect of Introduction Phase	Parent-Active Model (n = 210)				Traditional Model (n = 250)					Undefined Model (n = 75)						
	*M*	*SD*	*Min.* *Max.*		*M*	*SD*	*Min.* *Max.*			*M*		*SD*			*Min.* *Max.*	
Days allocated to introduction	7.77	3.09	1 15		9.71	2.89	3 20			8.91		2.58			3 15	
# children introduced simultaneously	2.86	2.02	1 9		2.32	1.93	1 11			1.77		1.07			1 5	
# teacher(s) assigned to each child	1.96	1.03	1 6		1.54	0.83	1 5			1.93		1.01			1 4	
h/day at preschool the first week	4.04	1.31	1 7.20		2.66	1.38	1 7			2.91		1.18			1 6	
**Preparatory Aspect of Introduction Phase**																
Background information about child/family before start	2.83	1.34	1 5		2.93	1.04	1 5			2.23		1.21		1 5		
Adjusting the introduction to child and family needs	4.28	0.85	2 5		4.25	0.76	2 5			4.09		0.78		2 5		
Child/family’s impact on the organization of introduction	3.65	0.91	2 5		3.55	0.95	1 5			3.61		0.98		1 5		
**Satisfaction with Introduction Conduct**																
Level of satisfaction with introduction model	4.29	0.94		1 5	4.45	0.72		1 5	4.03		1.06		1 5			

### Statistical Comparison of Organizational Characteristics of the Introduction Models

Univariate comparative analyses of the structural aspects of each model (Table 1) were conducted, including length and intensity of the introduction phase, number of children and teachers involved, and preparation.

#### Length and Intensity

Participants practicing the PAM reported allocating fewer days than those practicing the TM (*M diff =* 1.94; *H* (2) = 83.20; *p* < .0001; ε^2^ = 0.09) and UM (*M diff* = 1.14; *H* (2) = -44.59; *p* < .048; ε^2^ = 0.09). Similarly, families stayed about one and a half hour more per day at preschools practicing the PAM compared to those practicing the TM (*M diff =* 1.38; *H* (2) = -144.89; *p* < .001; ε^2^ = 0.21) and UM (*M diff =* 1.13; *H* (2) = 111.50; *p* < .001; ε^2^ = 0.21). A chi-square analysis demonstrated that the introduction models differed in timing of the first separation between child and parent, χ^2^(4, *n* = 527) = 23.37, *p* < .001, *V* = 0.15. Post hoc tests (Bonferroni corrected) indicated that it was more common (*n* = 142) to conduct the first separation at the end of the first week of the introduction phase compared to the second or third week (*n* = 27) when practicing the PAM. In contrast, for the TM, it was more common to conduct the first separation during the second or third week (*n* = 72).

#### Number of Children and Teachers

The TM assigned fewer teachers per introduced child than did PAM (*M diff* = 0,42; *H* (2) = -51.70; *p* < .001; ε^2^ = 0.05) or the UM (*M diff* = 0.39; *H* (2) = -48.77; *p* = .012; ε^2^ = 0.05). The PAM introduced about three children simultaneously, which was about one child more than the UM (*M diff* = 1.09; *H* (2) = 30.77; *p* = .035; ε^2^ = 0.05). Notably, 17% of participants, evenly distributed across the models, reported that the number of responsible teachers varied from time to time. About 64% reported that the number of children introduced simultaneously varied from time to time.

#### Preparation

Participants who practiced the UM obtained a lower amount of family background information before initiating the introduction than those practicing the TM (*H* (2) = 75.95; *p* < .001; ε^2^ = 0.03) and PAM (*H* (2) = 66.90; *p* = .003; ε^2^ = 0.03). However, no differences were found between the three models on how much they adjusted the introduction phase to the family and child’s needs or allowed the family and child to influence its organization.

#### Teachers’ Satisfaction with Introduction Conduct

Although the general level of satisfaction with the introduction conduct was overall high (*M* = 4.33; *SD* = 0.87), teachers practicing the TM were slightly (*M diff* = 0.42) but significantly (*H* (2) = 50.72; *p* = .015; ε^2^ = 0.02) more satisfied than those representing the undefined procedure.

### Descriptive Themes in Preschool Teachers’ Qualitative Accounts

The qualitative content analysis yielded six descriptive themes and eight sub-themes.

Quotations are marked with pseudonyms representing the respondent’s introduction model (TM, PAM, or UM). The relative frequencies of themes (i.e., number of participants that mentioned them) are reported in brackets. Participants commonly reported either one (57%) or two (26%) themes.

#### Theme 1: Relational Establishment (142 Participants; 34.38%)

This theme, consisting of codes such as “attachment”, “emotional security”, “relationship”, “contact”, “get to know”, and *“*trust”, conveyed that a satisfactory introduction phase depended on establishment of good relationships with the family. Good relationships were perceived as supporting the family’s sense of emotional security at the preschool, which helped the child’s adjustment.

### Subtheme 1.1: Child-Centered Relational Focus

When describing the importance of relational establishment during the introduction phase, participants referred to the necessity of interacting with both the child and parents: “We work hard to establish relationships with our new families” (PAM1). The reason was based on the perception that parents’ trust in the preschool was important for the child’s adjustment process. Nevertheless, they frequently mentioned directing the interactions specifically towards the child as a priority during the introduction process, as this helped form a connection specifically between the child and the teacher:

You can and should be frank and honest towards parents and talk about the fact that we first and foremost connect with and get to know the child, and due to this, we might not talk to the parents that much. (TM1)

Participants perceived these directed interactions as facilitating the child’s sense of emotional security: “I show the children a genuine interest of caring for them while their mum or dad is gone. They feel that I am an ‘extra’ mother to them” (UM1).

To enhance this process, it was common to assign one preschool teacher to be responsible for relationship-building with the child: “We assign one teacher as the responsible person for the introduction and to ensure that the child feels emotionally safe before the separation [from the parent]” (TM2).

#### Theme 2: Organizational Flexibility Towards the Needs of the Family (133 Participants; 32.20%)

This theme consisted of codes such as “flexible”, “adjustment”, “mindful of parents’/child’s needs”, “responsive to parents’/child’s needs”, and “different/individual needs”. Theme 2 illustrated that many preschool teachers associated a successful introduction with flexibly adjusting the process to meet the individual needs of the child and/or parent(s).

### Sub-theme 2.1: Allowing the Child to Lead the Way

Being responsive to children’s signals, rather than strictly following a pre-determined plan, was often mentioned as the essence of organizational flexibility: “I am happy that we have some degree of freedom to let the needs of the child set the agenda, rather than being forced to follow a pre-set document of conduct” (TM3).

Organizational flexibility was often regarded as adjusting the introduction length and/or timing of the first separation: “Sometimes there is a need to prolong the introduction [with more days] than originally planned, if pragmatically possible to the parents, of course. We have a plan we start out from, but it is flexible” (UM2).

Children’s preferences were sometimes used to determine which teacher would be responsible for the child’s introduction process: “We try to be sensitive towards the signals of the child, and we let the child ‘choose’ which teacher she/he feels most comfortable with” (UM3).

Adopting a flexible approach when introducing the child should not be understood as a lack of organizational clarity. Rather, it appears illustrative of fine-tuning practice as a “point of departure” for the introduction conduct.

#### Theme 3: Length, Intensity, and Child-Adult Ratio as Means of Success (88 Participants; 21.31%)

This theme, which contained codes like “long(er) period of time”, “short(er) days”, “no stress of finishing”, and “(too) many children”, illustrated how teachers’ perception of the success of the introduction phase was related to its structural aspects, such as length of introduction process and child–adult ratios.

### Subtheme 3.1: Letting Time Do the Trick

A long (10–15 days) introduction phase and having the child and parent at the preschool for a few hours per day was regarded as helpful for building relationships with the child: “I’m quite satisfied with the fact that we allocate two weeks to the introduction, as this gives me time to attach to the child and for the child to attach to me as a pedagogue” (TM4). Some participants also reported that more, but shorter days were beneficial for already established peer groups, being perceived as less invasive for everyday preschool life: “Many, short days are good for the children and do not affect the other preschool activities too much” (UM4).

### Sub-theme 3.2: More Than a Few Is a Crowd

About one fourth of the responses in this theme expressed concerns about group-introductions or conducting too many introductions during the school year: “I think that we introduce too many children simultaneously and put too little effort in the important bonding to the children” (PAM2). Too many introductions and large child–adult ratios were regarded as detrimental to teachers’ ability to constructively meet the individual needs of the family and connect relationally with each child.

#### Theme 4: Clearly and Consensually Defined Procedures (69 Participants; 16.71%)

The fourth theme described the importance of clearly defined procedures that are acknowledged and agreed on by all staff. It contained codes such as “cooperation”, “clear agenda/procedure”, “consensus”, “routines”, and “attuning expectations/approach” in relation to “teacher”, “staff team”, or “colleagues”.

### Subtheme 4.1: The Importance of Articulating the Implicit

Participants repeatedly emphasized the importance of sufficient time and opportunities to discuss and reflect on the rationale for the introduction activities. As one participant noted, “sensing” consensus is often insufficient:

I see that we have a consensus of what we do, but we don’t talk enough about it – for instance, we need to convey more clearly “the silent knowledge” to more inexperienced colleagues… They see what we do, but they may not always know why we do it. (UM5)

Clarity on the organizational structure was also considered necessary for better communication with parents about the content and purpose of the introduction phase: “Since there has not yet been time to brief [newly hired] staff about our routines [for introduction], they can’t convey the right information to the parents” (PAM3). The need to continuously articulate “what works and why” thus appears crucial if consensus on the model is to benefit the introduction process.

#### Theme 5: Actively Engaging the Parent (64 Participants; 16.71%)

The fifth theme, which contained codes such as “parental participation”, “active parent(s)”, “expectations on parents”, and “parent(s) at preschool”, described the importance of assigning the parent an active role during the introductory activities.

### Subtheme 5.1: “Emotionally Secure Parents Foster Emotionally Secure Children”

Engaging the parent actively was perceived to create opportunities for the parents to get to know the everyday life of the preschool and its teachers: “The parents are given good opportunities to see what our preschool activities look like, and they have the time to get to know us [preschool staff] and the preschool together with their child” (PAM4). This, in turn, indirectly benefited the emotional security of the child: “We work hard to make the parents feel emotionally secure with us and to have them actively show the child that the preschool is a secure and safe place. Emotionally secure parents foster emotionally secure children” (PAM5).

Actively engaging the parent also helped teachers to observe the specific needs of the child through the child–parent interactions:

As a teacher, I get to see how the child and its parent interact. All these small details that are otherwise never mentioned, such as the parent taking the child’s socks off, or caressing the child’s earlobe, when time for napping. (PAM6)

Ultimately, teachers’ reasons for actively involving the parent during the introduction process seemed to be the potential to support child–teacher bonding and, hence, the child’s adjustment process.

### Sub-theme 5.2: Lack of Time Ruling Out the Benefits of Active Parent Role

A few participants had reservations about an active parental role, as it sometimes shortened the rest of the introduction process:

I think that the parents are involved during the introduction for too few days (3–5 days before the first separation occurs). The child is not given enough time to establish a secure relationship to the teachers, and it becomes stressful to all of us. (PAM7)

One participant noted that actively involving parents allowed the preschool to introduce more children simultaneously, which they saw as problematic for child–teacher bonding as well: “I believe that the parent-active model, where many children are introduced simultaneously, was established from an economical perspective, to promote a quick intake of new children. I’m not sure if it is to the child’s best interest” (PAM8). Thus, conducting the first separation between the child and the parent after only few days of active parental involvement was seen as negatively impacting the chances for the child and teacher to bond.

#### Theme 6: Preparation of Family Before the Introduction (27 Participants; 6.54%)

Another component of a satisfactory introduction process was extensively preparing the family before enrolling the child, as illustrated by codes such as “preparation”, “information”, “meetings”, and “visits”.

### Subtheme 6.1: Using Preparation to Enable a Relational Focus

While some participants mentioned visiting the family’s home before initiating the introduction process, others made use of introduction meetings for parents. Preparing the family in advance on what to expect was seen as beneficial to teachers’ ability to focus on getting to know the child when initiating the introduction phase:

At the moment, questions and answers are provided at the beginning of the introduction phase, which doubles my task as I am forced to focus on both providing the parents with information and being present with the child when trying to establish contact. (TM6)

Other participants described that preparing helped the parents feel more relaxed about the situation when initiating the introduction process: “It [preparing parents] always works, and the parents feel emotionally secure as they know what will happen before they arrive” (TM7).

Making use of these preparatory activities before the introduction seemed, thus, to help preschool teachers and parents to focus on relating, rather than informing, during the actual introduction process.

#### Mixed Method Comparison of Descriptive Themes Across Introduction Models

Chi-square analyses were used to examine whether the six themes were related to specific introduction models (Table 2). Only theme 5 (“Actively engaging the parent”) differed across the introduction models. Multiple comparison tests with Bonferroni corrections indicated that it was more common for those practicing PAM to describe active parental involvement as an important factor of a successful introduction (χ^2^(2, *n* = 413) = 15.11, *p* = .001, *V* = 0.19).

**Table 2:** Distribution of Descriptive Themes in the Different Introduction Models

Theme	Parent-Active Model (Relative frequency)	Traditional Model (Relative frequency)	Undefined Model (Relative frequency)
Theme 1: Relational Establishment	52	76	14
Theme 2: Organizational Flexibility Towards the Needs of the Family	58	57	18
Theme 3: Length, Intensity, and Child-Adult Ratio as Means of Success	31	47	10
Theme 4: Cleary and Consensually Defined Procedures	31	31	7
Theme 5: Actively Engaging the Parent	40**	18**	6*
Theme 6: Preparation of Family Before the Introduction	5	17	5
*p < .05 **p < .0083			

## Discussion

According to both systems and attachment theories, the preschool transition is a developmentally essential context for the child; however, there is a lack of research that specify on how to conduct the introduction process. To our best knowledge, the present study is the first to describe and compare distinct models of preschool introduction based on their structural features, while also securing preschool teachers’ first-hand, evaluative accounts of the introduction process. This detailed charting of preschool introduction can offer a starting point for future research to investigate the effects of different preschool introduction strategies on families. We hope that this in turn can empirically inform how to organize interventions and strategies for introducing children to preschool.

### Distinct yet Flexible Organizational Features

We identified two distinct models of introduction: the TM (traditional model) and PAM (parent-active model). These findings expand and reinforce—through a statistically derived classification—previous qualitative evidence by Markström and Simonsson (2017). One departure from their findings was that group-introduction was a structural aspect of all introduction models in the present study, whereas Markström and Simonsson (2017) saw it as a distinct model.

The emergence of the UM—an undefined introduction procedure—suggests a worrisome lack of organizational clarity for introduction activities in some preschools. Well-structured procedures are considered characteristic of high quality preschools both in Sweden (The Swedish Schools Inspectorate, 2018) and internationally (Dennis & O’Connor, 2013). Indeed, compared to the TM and PAM, practicing the UM was associated with obtaining less background information on the child/family before initiating the introduction phase and with less satisfied teachers.

The small effect sizes and large variance for most structural aspects of the introduction models indicated a general degree of internal variability. This is less surprising for preschools without clearly defined procedures (UM) but is concerning for preschools with the PAM and TM because it can complicate the quality assurance of the procedures (e.g., Vermeer et al., 2016). While organizational changes related to COVID-19 (Andersson Søe et al., 2022) may have contributed to the low internal consistency, our results indicate that there is a degree of flexibility in the structural aspects of the introduction phase, regardless of introduction model (mentioned by 32.20% of the qualitative sample). This flexibility was described as a conscious and valuable organizational choice that helped in adapting the introduction process to the needs of each child and parent; thus, it is reasonable to conclude that there would be structural variations also outside of the pandemic.

#### Length and Intensity

The length and intensity of the introduction phase distinguished the models from each other. With the PAM, the child spent more hours per day at the preschool during the first week of the introduction, but fewer days were allocated to the introduction phase. These features statistically confirm previous qualitative descriptions of the PAM (Markström & Simonsson, 2017), albeit that the PAM described in this study could be longer than the three days suggested by Markström and Simonsson (2017). In fact, the qualitative analysis showed that teachers, regardless of introduction model, saw more but less intense days as facilitating child emotional adjustment and relationship-building. These findings suggest a flexible approach in relation to length and intensity when organizing the introduction.

Currently, there is little research on how length and intensity of preschool introduction influence children’s emotional adjustment and family–teacher relationship-building. Although the number of days for introduction does not appear to influence the perceived stress of the child (Ahnert et al., 2004), the presence of a parent for more days of the introduction phase may have a positively regulating effect on the child’s stress (Ahnert et al., 2004; Nystad et al., 2021). Thus, there may be value in postponing the first separation until the second or third introduction week, unlike the most common practice in the PAM. Indeed, some of those practicing the PAM expressed reservations about conducting the parent–child separation too soon, as a short introduction period also negatively affected their ability to bond with a child. While parental presence in itself, however, may only benefit the child’s adjustment if parents are able to emotionally regulate their child (Drugli, 2020), the amount of time that children spend with their parents during the introduction phase may still impact the opportunities for preschool teachers to interact and build relationships with the family.

### Relational Focus

Building relationships with the family (“Relational Establishment”) was the most frequently occurring theme in our qualitative analysis (mentioned by 34.38% of the qualitative sample) regardless of introduction model, which aligns with previous qualitative studies (Ebbeck & Yim, 2009; Hostettler Schärer, 2018). Teachers must balance this important relational focus on children’s need for socioemotional caregiving with didactic aspects of the pedagogical curriculum. However, the current tendency in preschool policy contexts is to emphasize teaching, both in Sweden (Ackesjö & Persson, 2019) and internationally (Schachter et al., 2021), and preschool teachers sometimes downplay aspects of their work that focus on relationship-building and caregiving to strengthen their professional status (Schachter et al., 2021). This is unfortunate, given the value assigned by attachment theory and prior research (e.g., Ahnert et al., 2006; Ulferts et al., 2019) to preschool teachers’ relational engagement with children (including their potential for strengthening the parent–child relationship; O’Connor et al., 2016). Parents have also expressed worry that caregiving is being under-prioritized due to the policy-backed focus on didactics (Van Laere et al., 2018). It does, however, not seem sufficient to merely call attention to teachers’ need to prioritize relational engagement with preschool children; rather, it may be necessary to more specifically assist them in *how* to organize the interactions with children to support their emotional adjustment (and in the long run, their secure relationships; Datler et al., 2012) in balance with their didactic role. The preschool introduction process is particularly important to focus on in this respect, as this is an arena for initiating the relationship-building (e.g., Brooker 2008).

Group-based introduction (i.e., introducing more than child) was, for instance, featured in all introduction models, but participants uniformly considered it problematic for child–teacher bonding. Although the precise effects of conducting large-group-based preschool introductions are unknown, limiting the number of children may be more conducive to individual bonding between the child and preschool teacher. However, child attachment development and emotional adjustment are not dependent solely on dyadic interactions between the child and specific caregivers; it may help to engage parents during the introduction process for this reason. Our finding that organization of parental participation differs across introduction models is thus of high theoretical and practical relevance for future study.

#### Emotionally Secure Parents Foster Emotionally Secure Children

Preparing parents before the introduction phase, such as by arranging visits at the preschool, providing practical information, or allowing opportunities for questions, was important in all introduction models. However, it was the least common theme in our qualitative results (mentioned by 6.54% of the qualitative sample). Repeated family-visits at the preschool before enrollment is considered advisable, since familiarizing the child with the preschool environment may facilitate their emotional adjustment during the transition process (Broström et al., 2016; Drugli, 2020). Several participants in this study described preparation as beneficial to the outcome of the introduction phase because it had a positive effect on parents’ attitudes towards the preschool, which in turn might promote child adjustment. In line with Markström and Simonsson’s study (2017), engaging parents actively in the introduction activities was statistically a significant feature of the PAM. However, although high parental engagement during preschool transition is considered advisable because of its potential benefit for child’s emotional adjustment (Ahnert et al., 2004; Nystad et al., 2021), there is little evidence of how to specifically make use of the parent(s) to this end. More importantly, we lack knowledge of how the parental role during preschool transition influences the child–teacher relationship-building.

The recently developed concept of “cocaring relationships” may be important to consider in this context (Lang et al., 2016, 2017). According to this concept, there is a triadic, reciprocal interconnectedness between child, parent(s), and preschool teacher in terms of relational quality, in line with findings that the quality of the parent–teacher relationship is linked to that of the child–teacher relationship (Chung et al., 2005; Jeon et al., 2021; Serpell & Mashburn, 2012). Accordingly, establishing a productive parent–teacher relationship during the introduction phase may be as important as child–teacher bonding. In this respect, as suggested by our results, actively engaging the parent(s) during preschool introduction may help parents and teachers get to know each other.

However, preschool teachers who shifted from actively engaging parents during the introduction process to a non-active parental role (due to COVID-19 distancing measures) reported more favorable relationship-building with children (Andersson Søe et al., 2022). We therefore suggest further investigation in future research of whether, and how, the parent–teacher and child–teacher relationships are related, and whether different ways of engaging the parent during preschool transition impact this triadic process. Exploring this may help inform how best to arrange the important parental participation during preschool introduction.

### Limitations

While collecting qualitative data through an open-ended survey design arguably results in less dense data, the “wide-angle lens” offered by this method (Braun et al., 2021), together with our mixed method analysis, enriched our results through the unique first-hand teacher perspective. Regarding our mixed methods analysis, it is worth mentioning that converting open-ended survey data into quantifiable categories does not create true dichotomous data, given that response options are endless. Thus, expressing one theme but not others should not be interpreted as active deselection of the unmentioned themes (Guest et al., 2012).

Despite efforts to ensure a systematic, credible qualitative analysis, we do not discount the influence of our views as researchers in our field of practice. We therefore continuously reflected on our subjectivity as researchers throughout the analysis to further strengthen the quality of the study design and results (Henwood & Pidgeon, 1992). The research team combines competences from the educational-psychological preschool field of practice with developmental-psychological perspectives in clinical settings. Although a background from the preschool field benefited the face-value of the study, it may have reduced the level of “open-mindedness” of the interpretations—however, this risk may have been mitigated through application of a developmental psychology perspective while also adding nuance to the findings.

While our sampling strategy yielded a large sample size with diverse characteristics, we cannot exclude the possibility of some self-selection in the sample. In addition, while several participants gave positive feedback on the relevance of the survey questions and pointed out that participation led to reflection, we cannot exclude the risk of social desirability bias. This is especially relevant to the questions on participants’ satisfaction with their preschool’s introduction conduct. However, our use of anonymous, online, open-ended survey questions might buffer the risk of social desirability (Braun et al., 2021). Moreover, we further reduced the risk by not asking participants for their preschool of employment.

## Conclusion

It is from a systems- and attachment developmental perspective essential to structure the transition process to preschool in a way that supports child emotional adjustment and family–teacher relationship-building. Our mixed methods approach enabled a large-scale, statistical assessment of how introduction conduct is structurally organized in Swedish preschools, integrating an analysis of an inductively derived teacher-perspective of “what works and why” during the introduction process. A particularly interesting contribution from the mixed methods analysis showed that different introduction models engaged parents differently during the introduction activities. When practicing the PAM, parents were engaged more actively in the introduction process, which may benefit parent–teacher relationship-building. This is important, as child adjustment in preschool over time might also be related to the quality of the parent–teacher relationship. Accordingly, introduction activities should not only focus on establishing relationships between the child and teacher, but also between parents and teachers. Yet, the presence of a parent for more days may better mitigate children’s emotional distress. This finding suggests it may be beneficial to employ a longer and less intense introduction phase, as in the TM. In the future, it is important to explore further whether, and how, parental participation affects child-parent-teacher interactions and parents’ ability to support their child’s adjustment during, and after, the introduction phase.

The nuanced classification of introduction models can inform future research in further investigations on how different organizational factors in preschool introduction might affect child adjustment and family–teacher relationship-building. However, as pointed out by Lamb & Ahnert (2007), different cultural understandings and legal prerequisites for childcare around the world complicate generalizability of the results of preschool research. Still, because parents and other primary caregivers are a natural part of preschool transition, our results, indicative of the importance to mind the parent–teacher relational quality when organizing preschool introduction, are relevant regardless of cultural background.
